# Fluorescence lifetime imaging reveals regulation of presynaptic Ca^2+^ by glutamate uptake and mGluRs, but not somatic voltage in cortical neurons

**DOI:** 10.1111/jnc.15094

**Published:** 2020-06-20

**Authors:** Olga Tyurikova, Kaiyu Zheng, Elizabeth Nicholson, Yulia Timofeeva, Alexey Semyanov, Kirill E. Volynski, Dmitri A. Rusakov

**Affiliations:** ^1^ Queen Square Institute of Neurology University College London London UK; ^2^ Shemyakin‐Ovchinnikov Institute of Bioorganic Chemistry Russian Academy of Sciences Moscow Russia; ^3^ Department of Computer Science, Centre for Complexity Science, University of Warwick Coventry UK; ^4^ Sechenov First Moscow State Medical University Moscow Russia

**Keywords:** axons, calcium imaging, neurotransmitter release, presynaptic mechanisms, synapse, synaptic plasticity

## Abstract

**Abstract:**

Brain function relies on vesicular release of neurotransmitters at chemical synapses. The release probability depends on action potential‐evoked presynaptic Ca^2+^ entry, but also on the resting Ca^2+^ level. Whether these basic aspects of presynaptic calcium homeostasis show any consistent trend along the axonal path, and how they are controlled by local network activity, remains poorly understood. Here, we take advantage of the recently advanced FLIM‐based method to monitor presynaptic Ca^2+^ with nanomolar sensitivity. We find that, in cortical pyramidal neurons, action potential‐evoked calcium entry (range 10–300 nM), but not the resting Ca^2+^ level (range 10–100 nM), tends to increase with higher order of axonal branches. Blocking astroglial glutamate uptake reduces evoked Ca^2+^ entry but has little effect on resting Ca^2+^ whereas both appear boosted by the constitutive activation of group 1/2 metabotropic glutamate receptors. We find no consistent effect of transient somatic depolarization or hyperpolarization on presynaptic Ca^2+^ entry or its basal level. The results unveil some key aspects of presynaptic machinery in cortical circuits, shedding light on basic principles of synaptic connectivity in the brain.

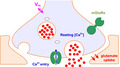

## Graphical abstract

Mechanisms of Ca^2+^‐dependent neurotransmitter release are key to understanding organisational principles of synaptic connectome. Monitoring presynaptic Ca^2+^ with nanomolar‐sensitive FLIM methods has revealed that, in cortical pyramidal neurons, spike‐evoked Ca^2+^ entry tends to increase with higher axonal branch orders. Blocking astroglial glutamate uptake reduces evoked Ca^2+^ entry with little effect on resting Ca^2+^ whereas both appear boosted by the constitutive activation of group 1/2 metabotropic glutamate receptors. We find no consistent effect of transient somatic depo‐ or hyperpolarisation on presynaptic Ca^2+^ entry or its basal level. The results unveil some principal aspects of presynaptic machinery in cortical circuits.

**Abbreviations**
2PE: two‐photon excitation
ANOVA: analysis of variance
FLIM: fluorescence lifetime imaging
HEPES: 4‐(2‐hydroxyethyl)‐1‐piperazineethanesulfonic acid
MES: measurement control and data analysis software
mGluR: metabotropic glutamate receptor
NMDG: N‐Methyl‐D‐glucamine diatrizoate
NTC: Normalized total count
OGB‐1: Oregon Green BAPTA‐1
RRID: research resource identifier
S‐MCPG: (s)‐(alpha)‐methyl‐4‐carboxyphenylglycine
SPCM: single photon counting modules
TBOA: DL‐Threo‐β‐Benzyloxyaspartic acid
TCSPC: Time‐correlated single‐photon counting

## 1 INTRODUCTION

Information processing and storage in the brain relies on Ca^2+^‐dependent release of the excitatory neurotransmitter glutamate from axonal terminals. Classical studies in preparations of giant synapses that enable direct experimental access, have revealed key mechanistic relationships between neurotransmitter release, evoked Ca^2+^ entry, and resting presynaptic Ca^2+^ (Bollmann, Sakmann, Gerard, & Borst, 2000; Eggermann, Bucurenciu, Goswami, & Jonas, 2012; Neher & Sakaba, 2008; Schneggenburger & Neher, 2000). In contrast, Ca^2+^ signalling at small central synapses, which are difficult to access in situ, has hitherto been explored mainly by monitoring the fluorescence intensity of Ca^2+^‐sensitive indicators. The intensity‐based approach has been instrumental in relating dynamic changes in presynaptic Ca^2+^ to use‐dependent plasticity of neurotransmitter release (reviewed in Regehr, 2012; Zucker & Regehr, 2002). However, intensity measures are prone to uncontrolled concomitants, such as changes in local dye concentration, photobleaching, tissue light scattering, or laser power fluctuations. These limitations could be critical for Ca^2+^ concentration ([Ca^2+^]) measurements whereas the accuracy of ratiometric Ca^2+^ indicators in optically turbid media, such as brain tissue, is compromised by the strong dependence between the wavelength and scattering/absorption of light. Thus, monitoring [Ca^2+^] inside individual axons, in particular the nanomolar range basal Ca^2+^ levels, has been a challenge.

A breakthrough came with exploring fluorescence lifetime sensitivity of some Ca^2+^ indicators to free Ca^2+^ (Wilms & Eilers, 2007; Wilms, Schmidt, & Eilers, 2006). As a time‐domain measure, fluorescence lifetime imaging (FLIM) is not influenced by light scattering, dye concentration, focus drift or photobleaching. We have recently advanced and validated an approach that optimizes FLIM‐based readout of such indicators in experimental settings in situ (Jennings et al., 2017; Zheng et al., 2015; Zheng, Jensen, & Rusakov, 2018). This method has enabled dynamic monitoring of presynaptic [Ca^2+^] in individual axons in situ, with nanomolar sensitivity (Jensen et al., 2019; Jensen, Zheng, Tyurikova, Reynolds, & Rusakov, 2017). Here, equipped with this approach, we asked, first, whether the excitatory synapses supplied by individual axons of cortical neurons show evenly distributed functional features of presynaptic Ca^2+^ signalling, or whether these features change along the axon. This quest has been an important line of enquiry into fundamental traits of circuit formation and function (Bakkum et al., 2013; Debanne, Guerineau, Gahwiler, & Thompson, 1997; Guerrero et al., 2005; Kukley, Capetillo‐Zarate, & Dietrich, 2007).

Second, we sought to understand whether and how the local excitatory activity affects presynaptic Ca^2+^. Glutamate released from axons is rapidly buffered and taken up, mainly by astroglial transporters (Danbolt, 2001): this keeps its extracellular concentration at the nanomolar level (Herman & Jahr, 2007) while limiting activation of intra‐ and extrasynaptic metabotropic glutamate receptors (mGluRs) (Huang & Bordey, 2004; Min, Rusakov, & Kullmann, 1998). Axons of cortical neurons often express group 2 mGluR2 and mGluR3, but also group 1 mGluR1 and mGluR5 subtypes of mGluRs (Cartmell & Schoepp, 2000; Gereau & Conn, 1995), with recent evidence implicating group 2 mGluRs in presynaptic inhibition in human cortex pyramidal cells (Bocchio et al., 2018). These two receptor subgroups enable cellular cascades that trigger, respectively, either inhibition or mobilization of presynaptic Ca^2+^ signalling (Cartmell & Schoepp, 2000; Pinheiro & Mulle, 2008; Reiner & Levitz, 2018). The net effect of such signalling, in terms of presynaptic [Ca^2+^] changes, remains poorly understood.

Finally, our aim was to establish whether somatic depolarization (or hyperpolarization) of the host neuron affects its axonal Ca^2+^ signalling. This issue has long been a subject of debate. It has been shown that depolarizing central neurons can boost glutamate release from distant axonal boutons (Alle & Geiger, 2006; Christie, Chiu, & Jahr, 2011; Scott, Ruiz, Henneberger, Kullmann, & Rusakov, 2008; Shu, Hasenstaub, Duque, Yu, & McCormick, 2006). However, axonal Ca^2+^ imaging (using fluorescence‐intensity measures) has suggested that, in hippocampal granule cells, somatic depolarization reduces spike‐evoked presynaptic Ca^2+^ entry in proximal axonal segments (Ruiz et al., 2003; Scott et al., 2014) while having no detectable effect distally (Scott et al., 2008). In contrast, in cortical pyramidal cells, somatic depolarization was proposed to boost spike‐evoked presynaptic Ca^2+^ entry (Christie et al., 2011; Shu et al., 2006) whereas it was presynaptic hyperpolarization that enhanced transmission between cortical or hippocampal pyramidal cells (Rama et al., 2015). The role of the underlying Ca^2+^ mechanisms has therefore remained debateable, mainly because of the limitations imposed by the traditional fluorescence intensity‐based Ca^2+^ measures. We therefore thought that it was important to explore the FLIM‐based approach, in the context.

## 2 MATERIALS AND METHODS

### 2.1 Animal experimentation

All experiments involving animals were carried out in accordance with the European Commission Directive (86/609/EEC) and the United Kingdom Home Office (Scientific Procedures) Act (1986) under the Home Office Project Licence PPL P2E0141 E1. C57BL/6 mice (Charles River Laboratories; IMSR Cat#JAX_000664, RRID: IMSR_JAX: 000664) of both sexes (60% male and 40% female) were group housed in a controlled environment as mandated by the locally approved guidelines, on a 12 hr light cycle and with food and water provided *ab libitum*. This study was not pre‐registered.

### 2.2 Brain slice preparation

Acute 300 μm thick coronal brain slices were obtained from 47 3–4 week old C57BL/6 mice (15–20 g), in full compliance with national guidelines on animal experimentation, in accord with Schedule I humane procedures. Animals were anaesthetized by 5% isoflurane inhalation, deep anaesthesia was ensured by a lack of pedal reflexes; after cessation of breathing animals were decapitated for brain isolation and removal. The locally approved isoflurane anaesthesia is sufficiently potent to provide muscle relaxation adequate for ascribed procedure and produces less cerebral vasodilation than analogues (e.g. by halothane); absorption and elimination of isoflurane inhalation occur through the lungs and allow rapid and reliable aesthetic induction. Slices were prepared in an ice‐cold slicing solution containing (in mM): NMDG, 92 (Sigma‐Aldrich; Cat#M2004); KCl, 2.5 (Sigma‐Aldrich; Cat#60130); NaH_2_PO_4_, 1.25 (Sigma‐Aldrich; Cat#S8282); HEPES, 20 (Sigma‐Aldrich; Cat#54457); thiourea, 2 (Sigma‐Aldrich; Cat#PHR1758); sodium ascorbate, 5 (Sigma‐Aldrich; Cat#PHR1279); sodium pyruvate, 3 (Sigma‐Aldrich; Cat#P8574); MgCl_2_, 10 (Sigma‐Aldrich; Cat#63069); D‐glucose, 25 (Sigma‐Aldrich; Cat#G8270); NaHCO_3_, 30 (Sigma‐Aldrich; Cat#S6297); CaCl_2_, 0.5 (Sigma‐Aldrich; Cat#21115); and sucrose, 1 (Sigma‐Aldrich; Cat#S0389). For recovery slices were left for 15–20 min in slicing solution and for 40 min at 34°C ACSF solution, before being transferred to a submersion chamber for storage in an extracellular solution containing (in mM): NaCl, 125 (Sigma‐Aldrich; Cat#S7653); KCl, 2.5; NaH_2_PO_4_, 1.25; NaHCO_3_, 26 (Sigma‐Aldrich; Cat#S6297); D‐glucose, 18; CaCl_2_, 2; MgSO_4_, 1.3 (Sigma‐Aldrich; Cat# 63138)(osmolarity adjusted to 295–310 mOsM with D‐glucose). All solutions were continuously bubbled with 95% O_2_/5% CO_2_. Slices were allowed to rest for at least 60 min before recordings started.

### 2.3 Electrophysiology, axon tracing and Tornado scanning in pre‐synaptic boutons

We used a Femto2D‐FLIM two‐photon excitation (2PE) imaging system (Femtonics, Budapest), integrated with patch‐clamp electrophysiology (Scientifica, UK) and optically linked to two femtosecond pulse lasers MaiTai (SpectraPhysics‐Newport), with independent shutter and intensity control. Patch pipettes were prepared with borosilicate–standard wall filament glass (G150F‐4; Warner Instruments), with 4–5 mOsM resistance. Internal solution contained (in mM): KCH_3_O_3_S, 130 (Sigma‐Aldrich; Cat#83000); NaCl, 8; HEPES, 10; phosphocreatine disodium, 10 (Sigma‐Aldrich; Cat#P7936); Na_2_GTP, 0.4 (Sigma‐Aldrich; Cat#10106399001); MgATP, 4 (Sigma‐Aldrich; Cat#A9187); sodium ascorbate, 3 (pH‐adjusted to 7.2 with KOH; osmolarity‐adjusted to 290–295 mOsM), and supplemented with the morphological tracer dye Alexa 594 (50 μM; Thermo Fisher Scientific; Cat#A10438) with addition of Oregon Green BAPTA‐1 (300 μM; Thermo Fisher Scientific; Cat# O6807) for FLIM recordings. Following whole‐cell break‐in, 40–60 min were allowed for the dyes to equilibrate across the cell, and then the axonal arbour was traced in frame‐scan mode, also using z‐axis browsing, until the first axonal bouton had been identified as described previously (Jensen et al., 2017). Pre‐synaptic imaging was carried out in current clamp mode (*V*_m_ ≈ −70 mV) using an adaptation of pre‐synaptic Ca^2+^ imaging methods previously described (Jensen et al., 2017, 2019). Cortical neurons requiring compensation current of > 70 pA were discarded before imaging. In the imaging channels, cells demonstrating trial‐to‐trial fluctuations in the baseline [Ca^2+^] or evoked [Ca^2+^] over ~20% were discarded. Once the bouton was identified, position and size of spiral shaped (Tornado) line scans were adjusted to cover the visible bouton profile, and recorded as described below. Depending on the bouton size, one spiral scan typically takes 1–1.5 ms, thus providing readout of axonal fluorescence with high temporal and spatial resolution. Individual action potentials were evoked by a 2 ms pulse of depolarizing current (0.9–1.5 nA), in current clamp mode, as detailed previously (Scott et al., 2014).

### 2.4 2PE Tornado‐FLIM readout of Ca^2+^ concentration in small axonal boutons

In slice preparations, we thus identified and patched pyramidal neurons located in layer 2/3 of the visual cortex. Cell axons were followed, as described above, to focus on individual boutons; during individual trials (typically lasting 2 s), continuous tornado line scans were collected. The scan data were recorded by the standard analogue integration in Femtonics MES (RRID: SCR_018309), and by TCSPC in Becker and Hickl SPCM (RRID: SCR_018310) using dual HPM‐100 hybrid detectors. Next, we used the fast‐FLIM analysis procedure described previously (Zheng et al., 2015, 2018) to handle individual Tornado scans. We routinely collected and stored FLIM line scan data in a *t × x × y × T* data cube representing an *x‐y* image with the distribution of nanosecond decay timestamps (*t*) of individual photons, pixel‐by‐pixel over the frame duration (*T*). However, for the purposes of this study, we collapsed all spatial information thus boosting photon counts per scan cycle. The FLIM data represented therefore the average signal over the bouton area (approximately the entire profile) covered by the scan. Post‐hoc FLIM analyses were performed in a custom‐made data analysis toolbox, which is available online (https://github.com/zhengkaiyu/FIMAS; RRID: SCR_018311). The fluorescence decay curve (lifetime photon counts) was integrated over the 9 ns period post‐pulse, and normalized to the maximum value, as detailed earlier (Zheng et al., 2018). Data from up to 5–10 neighbouring pixels were averaged to ensure that the FLIM decay traces had sufficient counts towards the tail of the decay (8–12 ns post‐pulse). Data from a single trial were normally sufficient for boutons located closer to the surface of the tissue; for deeper‐located boutons, several trials were required to estimate accurately the Ca^2+^ dynamics evoked by an AP.

### 2.5 Estimating action potential evoked presynaptic Ca^2+^ entry

The (steady‐state) basal presynaptic [Ca^2+^]_0_ was directly estimated from FLIM readout over the averaging interval of ~500 ms before an action potential. However, the rapid rise of presynaptic [Ca^2+^] (1–2 ms) was faster than the averaging time of FLIM recording (5–10 ms). Therefore, to improve the signal‐to‐noise ratio in measuring presynaptic Ca^2+^ entry Δ[Ca^2+^], the spike‐evoked peak presynaptic [Ca^2+^]_peak_ was estimated using both FLIM and intensity recordings as follows. First, the saturated OGB‐1 fluorescence value *F*_max_ was estimated as $F_{\max}=F_{\text{rest}}\frac{{\left[{\text{Ca}}^{2+}\right]}_{0}+K_{d}}{{\left[{\text{Ca}}^{2+}\right]}_{0}+\left(K_{d}/\gamma \right)}$ where [Ca^2+^]_0_ is measured directly with FLIM, and *K*_d_ = 0.24 µM and γ = 6 are Ca^2+^ affinity and dynamic range of OGB‐1, respectively (Scott & Rusakov, 2006). Second, [Ca^2+^]_peak_ (equilibrated over 1–2 ms) was calculated as ${\left[{\text{Ca}}^{2+}\right]}_{\text{peak}\;}=K_{d}\frac{F_{\text{peak}}-\left(F_{\max}/\gamma \right)}{F_{\max}-F_{\text{peak}}}$ (Maravall, Mainen, Sabatini, & Svoboda, 2000; Tsien, 1989), so that Δ[Ca^2+^] = [Ca^2+^]_peak_ – [Ca^2+^]_0_. As an extra validation step, the fluorescence intensity decay was checked for a match with the FLIM readout decay, in the linear range of OGB‐1 sensitivity to [Ca^2+^].

### 2.6 Statistical analysis

During axonal tracing with 2PE imaging, axonal boutons were sampled in an arbitrary manner, as they appeared in the focal plane showing distinct varicose morphology and clear action potential induced Ca^2+^ responses. No exclusion criteria were applied to animals or slices; unhealthy patched cells were excluded according to the criteria described above. Blinding was not applicable to experimental manipulations during live recording. Thus no strict randomization procedures were applicable during 3D axonal tracing. In experiments comparing independent samples in control condition (branch order comparisons), both two‐way ANOVA and conservative non‐parametric Kruskal–Wallis ANOVA tests were applied as described. In the real‐time experiments involving application of a ligand or a voltage change, the statistical unit was individual boutons, with the effect of experimental manipulation being the only factor of interest; normally, 1–4 boutons were recorded from individual cells, 1–2 cells were recorded per slice/ animal. The paired 'baseline‐effect' comparison was therefore employed in all such experiments, in accord with the electrophysiological convention. The sample size was not predetermined because the variability of measured parameters was not known a priori. Shapiro–Wilks tests for normality produced varied results across raw data samples. Accordingly, we used either the paired‐sample *t*‐test, or the paired‐sample non‐parametric Wilcoxon Signed Ranks test, as indicated. The statistical software in use was Origin 2019 (Origin Lab; RRID: SCR_014212).

## 3 RESULTS

### 3.1 Monitoring presynaptic [Ca^2+^] using FLIM‐based readout

**FIGURE 1:**
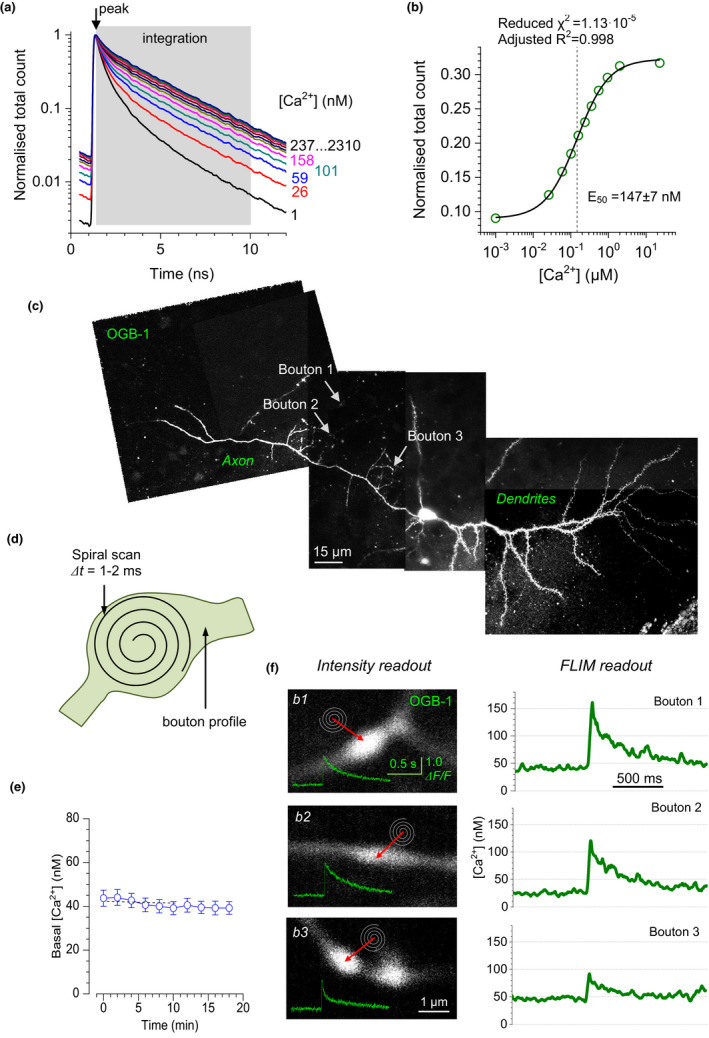
Two‐photon excitation FLIM‐based monitoring of intracellular [Ca^2+^] in small axonal boutons in cortical neurons in situ. (a) Calibration of FLIM readout in clamped calcium solutions using a femtosecond pulse infra‐red laser (100–200 ps pulse, λ_x_^2p^ = 800 nm); fluorescent decay traces post pulse, normalized to the peak (arrow), in a series of clamped [Ca^2+^] solutions, as indicated (and colour coded); shaded area indicates integration (area‐under‐the‐curve) interval, which is related to the peak value (arrow); experiments at 33°C. (b) Normalized total photon counts (circles) obtained from fluorescence decay plots as in (a), plotted against log[Ca^2+^]; the data are fitted with a logistic function (solid line), as indicated, showing E_50_ value for free [Ca^2+^]. (c) Tracing small axonal boutons of a layer 2/3 pyramidal neuron patched in whole‐cell (Alexa channel, λ_x_^2p^ = 800 nm) in an acute slice of a ~4wo mouse. Image collage (z‐averaged 3D‐stacks of focal‐plane images collected at different parts of the cell); whole‐cell mode (with 300 µM OGB‐1); 3D‐reconstructed dendritic and axonal branches are indicated (z‐axis projection); three axonal boutons (Boutons 1–3) are selected for imaging, as shown. (d) A schematic illustrating the application of rapid spiral line‐scanning in axonal bouton imaging; spiral line, repeated trajectory of the focused laser beam. (e) A FLIM measure stability test, showing basal [Ca^2+^] readout (mean ± *SEM*, *n* = 32 boutons, 17 animals) in control conditions calculated during 10 repeated cycles of measurement (2 min apart), as indicated. (f) Images, three boutons (*b1‐3*) selected for imaging as shown in (c), with the spiral scan positioning as illustrated (centred at the arrowhead); inset traces, a characteristic OGB‐1 fluorescence intensity response to an action potential. Plots, presynaptic [Ca^2+^] time course, reconstructed from FLIM data, upon generation of an action potential at the soma. Note that such data report [Ca^2+^] values that are space‐time equilibrated over 1–2 µm^3^ (approximate point‐spread function volume) and 2–3 ms (time for diffusion equilibration across the bouton), and necessarily time‐averaged by FLIM acquisition over ~7 ms steps

To calibrate FLIM readout for absolute [Ca^2+^] measurements on a designated two‐photon excitation (2PE) microscopy imaging system, we employed the protocol established for OGB‐1 previously (Zheng et al., 2015, 2018). The procedure uses the ratiometric Normalized Total Count (NTC) method in which photon counts are integrated under the lifetime decay curve (over its Ca^2+^‐sensitive span), and the result is related to the peak value (Materials and Methods; Figure 1a). The outcome confirmed high sensitivity of the readout in the 0–300 nM [Ca^3+^] range, providing a quantitative reference to the microscopy measurements (Figure 1b). This calibration outcome was similar to the data set obtained previously for a different 2PE system (Zheng et al., 2015, 2018), arguing for the robustness of the present approach.

We next held individual layer 2/3 pyramidal cells in whole‐cell mode dialysing them with 300 µM OGB‐1, and traced their axons up to a distance of 250–300 µm from the soma, in two‐photon excitation (2PE) mode (Figure 1c). Once focussed on individual axonal boutons, we used spiral (tornado) line scan (at 500–1000 Hz) covering the bouton profile (Figure 1d), to record Ca^2+^‐sensitive photon count data, before and after triggering a somatic spike (Jensen et al., 2017, 2019). With the averaging of the spatial scan data (Methods), this type of recording provides stable photon count acquisition from a small region of interest during repeated trials over ~20 min (Figure 1e). This was consistent with the previously documented FLIM recording stability, in similar settings, for up to 60 min (Jensen et al., 2019; Zheng et al., 2018). Thus, decoding the recorded FLIM data provided robust traces of resting basal [Ca^2+^]_0_ and spike‐evoked presynaptic [Ca^2+^] dynamics, in the 10–300 nM range, for boutons located at axonal branch orders 1–3, at different distances from the soma (Figure 1f).

### 3.2 Resting Ca^2+^ and evoked Ca^2+^ entry change with axonal branch order

**FIGURE 2:**
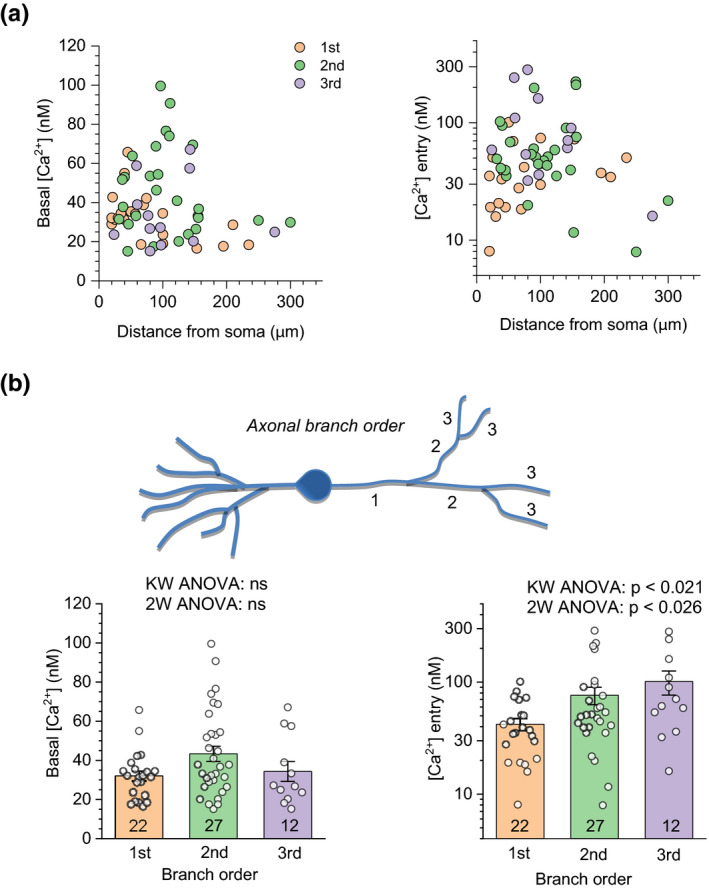
Presynaptic baseline Ca^2+^ level and evoked Ca^2+^ entry tend to increase with higher branch order. (a) Individual values of basal presynaptic [Ca^2+^] ([Ca^2+^]_0_, left) and spike‐evoked Ca^2+^ entry (Δ[Ca^2+^], right, *y*‐axis log scale) in presynaptic axonal boutons of pyramidal cells, at different branch orders and distances from the soma, as indicated. See Methods for measurement detail; individual data points may have a measurement error of several nM, because of limited photon count in small structures (Zheng et al., 2018). (b) Inset, an illustration of axonal branch order numbers 1–3. Graphs, summary of [Ca^2+^]_0_ and Δ[Ca^2+^] data grouped with respect to the axonal branch order. Individual bouton data (circles) and average values (bars ± *SEM*) of [Ca^2+^]_0_ (left; mean ± *SEM*: 31 ± 2, 45 ± 4, and 34 ± 5 nM; *n* = 22, 27, 12 boutons recorded from 13, 13, 9 animals respectively) and Δ[Ca^2+^] (right, log scale; 42 ± 5, 76 ± 13, and 101 ± 24 nM, respectively) are shown. Statistical significance of difference because of branch order (*P* value) was tested using Kruskall–Wallis (KW) ANOVA (branch‐order factor, *df* = 2); and two‐way ANOVA (2W ANOVA; branch‐order factor, *df* = 2; individual cell factor, *df* = 24), as indicated

We thus collected data on resting presynaptic [Ca^2+^] ([Ca^2+^]_0_) and spike‐evoked Ca^2+^ entry (concentration increment Δ[Ca^2+^]) from 61 axonal boutons in 25 pyramidal cells. First, the results indicated no overall dependence of either [Ca^2+^]_0_ or Δ[Ca^2+^] on the distance from the soma (Figure 2a). This data set uncovered significant heterogeneity of both [Ca^2+^]_0_ (detected range ~10–100 nM) and, especially, Δ[Ca^2+^] (detected range ~10–300 nM) across the axonal population (Figure 2a). Comparing bouton populations representing a certain order of axonal branches (from one to three, Figure 2b inset) revealed apparent trends (Figure 2b graphs). To understand whether these trends were significant, we ran ANOVA analyses. Because the Shapiro‐Wilks test for normality gave varied results across the branch‐order nested samples (normality was rejected in four out of six cases), we first ran the non‐parametric Kruskal‐Wallis ANOVA, with the branch order as a single factor, and second a two‐way ANOVA with the branch order and the cell identity as two factors. These two approaches produced consistent results, indicating that the axonal branch order had no significant overall influence on [Ca^2+^]_0_ (Figure 2b, left; here [Ca^2+^]_0_ was affected by the factor of cell identity, *p* < .001), but had an effect on Δ[Ca^2+^] (where individual cells had no significant effect) (Figure 2b, right). These data indicate that some basic features of presynaptic Ca^2+^ homeostasis are distributed along cortical cell axons heterogeneously, with higher order branches, rather than greater distances from the soma, favouring stronger evoked Ca^2+^ entry.

### 3.3 Glutamate uptake and metabotropic glutamate receptors differentially affect [Ca^2+^]_0_ and Δ[Ca^2+^]

**FIGURE 3:**
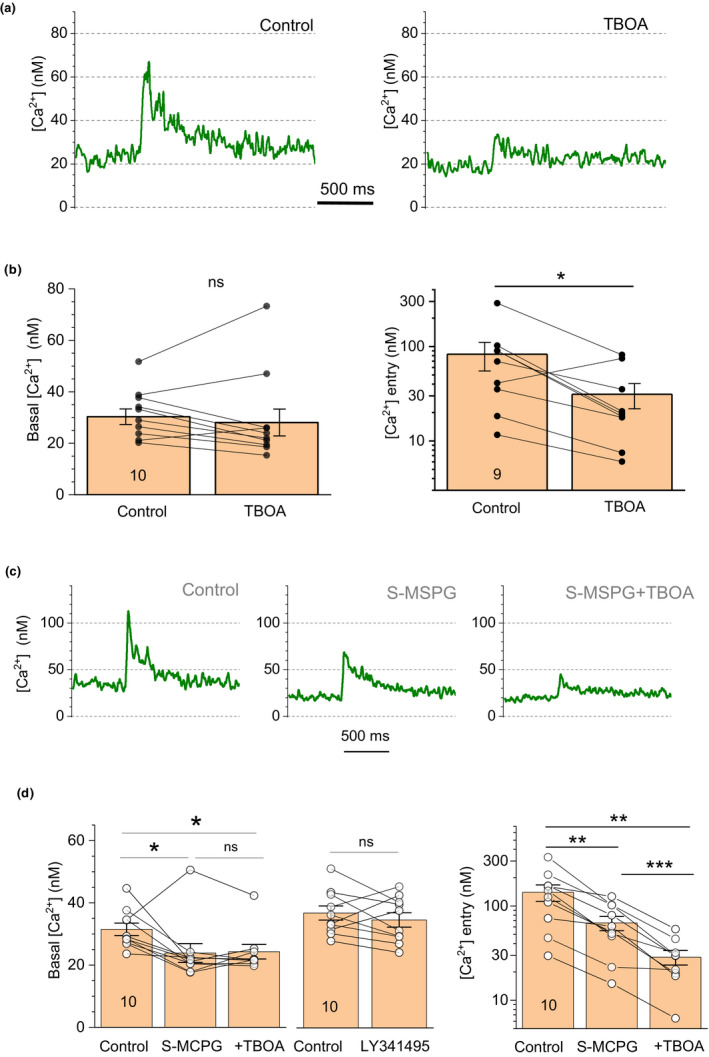
Glutamate uptake and metabotropic glutamate receptors differentially control presynaptic Ca^2+^ homeostasis. (a) Characteristic time course of presynaptic [Ca^2+^] dynamics (FLIM‐readout) in baseline condition (left) and 15 min after glutamate transporter blockade with 50 µM TBOA (right): one‐bouton example. (b) Summary of experiments (individual data points and mean ± *SEM*) shown in (a), for average values of basal Ca^2+^ level [Ca^2+^]_0_ (left: 30 ± 3 and 28 ± 5 nM in control and TBOA, respectively, *n* = 10 boutons, 4 animals), and spike‐evoked Ca^2+^ entry (Δ[Ca^2+^], right, log scale: 99 ± 22 to 31 ± 6 nM, in control in TBOA, respectively, *p* < .05, *n* = 9 boutons, 4 animals); *p < .03 (Paired‐sample Wilcoxon Signed Ranks Test; normality of data scatter rejected). Lines connect data points from the same bouton. (c) Characteristic time course of presynaptic [Ca^2+^] dynamics (FLIM‐readout) in baseline condition (left), 15 min after application of 200 µM S‐MCPG (middle), and 15 min after subsequent transporter blockade with 50 µM TBOA (right): one‐bouton example. (d) Summary of experiments (individual data points and mean ± *SEM*) shown in (c), also including group 2 mGluR inhibition. *Left:* Average values of basal Ca^2+^ level [Ca^2+^]_0_ (mean ± *SEM*): 31 ± 1, 23 ± 3, and 24 ± 2 nM, in control, S‐MSPG, and added TBOA, respectively (*n* = 10 boutons, 4 animals); 36 ± 2 and 34 ± 2 nM, in control and LY341495, respectively (*n* = 10 boutons, 1 animal). *Right*: Average spike‐evoked Ca^2+^ entry Δ[Ca^2+^] (*y*‐axis log scale): 140 ± 28, 66 ± 11, and 29 ± 5 nM in S‐MSPG, and added TBOA, respectively (*n* = 10 boutons, 4 animals); *p < .05, **p < .01, *** *p* < .005 (paired *t*‐test, normality not rejected). Lines connect data points from the same bouton

To understand whether and how glutamate uptake affects presynaptic Ca^2+^ signalling, we documented changes in [Ca^2+^]_0_ and Δ[Ca^2+^] in response to the pharmacological blockade of astroglial glutamate transporters. Application of the transporter inhibitor TBOA (Tsukada, Iino, Takayasu, Shimamoto, & Ozawa, 2005) had no detectable effect on [Ca^2+^]_0_ while depressing spike‐evoked Δ[Ca^2+^] by ~70% (Figure 3a and b). This suggests that the extracellular glutamate level elevated by TBOA application can inhibit Ca^2+^ entry through presynaptic Ca^2+^ channels, either through an ionotropic (electrogenic) mechanism, such as membrane depolarization, or through the action of presynaptic metabotropic glutamate receptors, or both. To distinguish between these two mechanisms, we recorded [Ca^2+^]_0_ and Δ[Ca^2+^] in individual axonal boutons in baseline conditions, 15 min after washing in the selective group 1/2 mGluR blocker S‐MCPG, and 15 min after the subsequent application of TBOA.

S‐MCPG application reduced [Ca^2+^]_0_ by ~ 25% (Figure 3c and d, left), suggesting that group 1 or group 2 mGluRs, by being persistently (constitutively) activated, contribute an additional Ca^2+^ source to the equilibrated presynaptic basal Ca^2+^. To distinguish between the two receptor subtypes, we repeated these tests with the specific group 2 mGluR blocker LY341495 and found no effect on [Ca^2+^]_0_, thus indicating the prevalent role of group 1 mGluR in the constitutive control of [Ca^2+^]_0_. The blockade of glutamate transporters in the presence of S‐MCPG had little further effect on [Ca^2+^]_0_, consistent with no effect of TBOA in control conditions (Figure 3b, left). The fact that boosting the extracellular glutamate level has no effect on [Ca^2+^]_0_ (Figure 3b, left) whereas blocking mGluRs reduces it (Figure 3d, left) suggests that, firstly, constitutive activation of group 1 mGluRs does not depend on glutamate and, secondly, once glutamate‐activated, the receptor suppresses evoked Ca^2+^ entry (Figure 3b, right). However, S‐MCPG application did reduce Δ[Ca^2+^] by ~ 50%, which was further depressed by TBOA (Figure 3d, right). This result suggests, firstly, that the TBOA‐induced decrease in Δ[Ca^2+^] (Figure 3b, d; right) does involve group 1/2 mGluRs. Secondly, it relates constitutive activation of these receptors to increased evoked presynaptic Ca^2+^. In our tests, the effect of the specific group 2 mGluR blocker LY341495 on Δ[Ca^2+^] was inconclusive as the cells became unstable during spike initiation (see Discussion). Overall, these findings may reflect a complex nature of presynaptic Ca^2+^ control by different mGluR subtypes (see Discussion).

### 3.4 Subthreshold somatic depolarization (or hyperpolarization) has no consistent effect on [Ca^2+^]_0_ or Δ[Ca^2+^]

**FIGURE 4:**
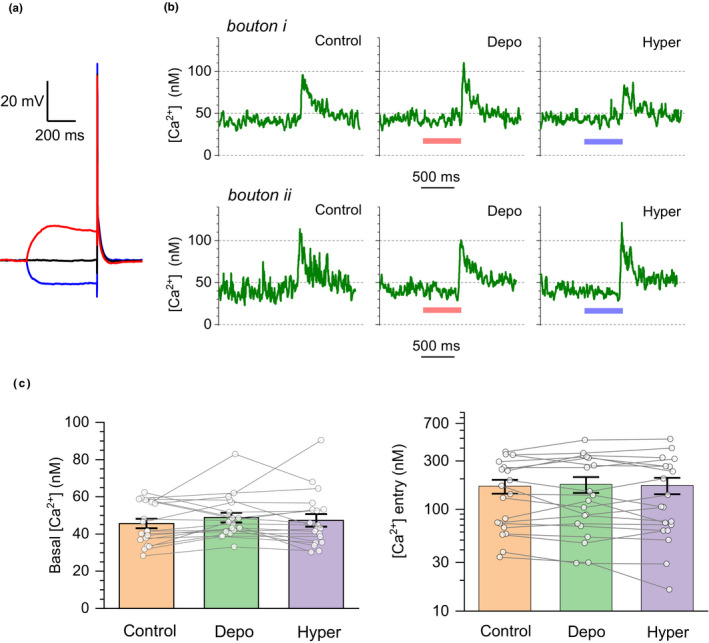
Somatic membrane potential has little influence on presynaptic Ca^2+^ dynamics in cortical neurons. (a) Example of whole‐cell (current clamp) recording trace illustrating three conditions: baselines (black), 500 ms depolarization pulse (red, approximately + 15 mV), and 500 ms hyperpolarization pulse (blue, approximately −15 mV) applied prior to the evoked action potential, in current clamp configuration. (b) Characteristic time course (two‐bouton example) of presynaptic [Ca^2+^] (FLIM readout) in the three conditions, as indicated, for two individual axonal boutons; colour bars indicate period of somatic depolarisation (red) and hyperpolarization (blue). (c) Summary of experiments shown in (a and b): dots, individual boutons; bars, mean ± *SEM*. *Left:* Average values of basal Ca^2+^ level [Ca^2+^]_0_ (mean ± *SEM*: 46 ± 2, 49 ± 3, and 47 ± 3 nM, in control, depo‐, and hyperpolarization conditions, respectively; *n* = 19 boutons, 8 cells, recorded from three animals). *Right:* Average spike‐evoked Ca^2+^ entry Δ[Ca^2+^] (log scale: 169 ± 26, 176 ± 31, and 173 ± 32 nM in control, depo‐, and hyperpolarization conditions, respectively; *n* = 19 boutons, 8 cells, from three animals). Lines connect data points from the same bouton

To understand the effect of somatic depolarization on presynaptic Ca^2+^ dynamics, we documented [Ca^2+^]_0_ and Δ[Ca^2+^] in individual axonal boutons when the presynaptic cell was either depolarized, or hyperpolarized, by ~15 mV either way for 500 ms prior to evoking an action potential (Figure 4a). In each selected axonal bouton, all three conditions were tested in an arbitrary sequence, to avoid any longer term effects. Overall, we found no consistent effect of somatic voltage manipulation on either [Ca^2+^]_0_ or Δ[Ca^2+^] in *n* = 19 boutons recorded in eight pyramidal cells (Figure 4b and c).

## 4 DISCUSSION

In the present study, we employed an imaging method that could detect changes in presynaptic [Ca^2+^] with virtually nanomolar sensitivity in the concentration range between 10‐300 nM (Zheng et al., 2015, 2018). We have documented average [Ca^2+^]_0_ values in baseline conditions between 30 and 60 nM, which is consistent with earlier high‐sensitivity Ca^2+^ measurements in neuronal processes (Canepari, Vogt, & Zecevic, 2008; Helmchen, Imoto, & Sakmann, 1996), including axons (Ermolyuk et al., 2013), that employed alternative Ca^2+^ imaging methods. Similarly, the range of Δ[Ca^2+^] between 50 and 300 nM reported here corresponds to the equilibrated presynaptic [Ca^2+^] after a very brief (~1 ms) local 'hotspot' entry, and is fully in line with previous estimates based on fluorescence‐intensity measures (Ermolyuk et al., 2013; Helmchen et al., 1996; Rusakov, Saitow, Lehre, & Konishi, 2005; Scott & Rusakov, 2006). However, the FLIM‐based method has several advantages over previous approaches, which enables us to explore presynaptic [Ca^2+^] dynamics in greater detail, as discussed earlier (Wilms et al., 2006; Zheng et al., 2018; Zheng & Rusakov, 2015).

The quest to identify a systematic pattern of functional synaptic features along the axon has been an important line of enquiry into fundamental traits of circuit formation and function (Bakkum et al., 2013; Debanne et al., 1997; Guerrero et al., 2005; Kukley et al., 2007). One of the most common questions asked in this context has been whether the increasing sparsity of longer cell‐cell connections in the cortex is compensated by their increased synaptic efficacy. We have recently employed multiplexed imaging of glutamate release and presynaptic Ca^2+^ in organotypic brain slices to find that [Ca^2+^]_0_ and Δ[Ca^2+^] are positively correlated with release probability (Jensen et al., 2019). Thus, the present data appear to argue against increased release efficacy with greater distances from the soma, but they do support the idea that in cortical pyramidal cells, axonal branches of higher orders host more efficient release sites (Figure 2). Clearly, imaging glutamate release at individual axonal boutons should provide further clarity on the subject. However, no known time‐resolved (FLIM‐based) optical sensors of glutamate are available at present. Therefore, to gauge accurately glutamate release efficacy in the turbid medium of acute cortical slices or in vivo, a special effort would be required to avoid multiple concomitants of the fluorescence intensity signal, for its unbiased interpretation.

We have found that the blockade of the group 1 mGluRs, which occur in cortical axons (Cartmell & Schoepp, 2000; Gereau & Conn, 1995), reduces presynaptic basal [Ca^2+^], suggesting that these receptors are constitutively active, in a glutamate‐independent manner. These receptors are known to trigger a powerful molecular cascade initiating local IP_3_‐receptor dependent release from Ca^2+^ stores, both in neurons (Pinheiro & Mulle, 2008; Reiner & Levitz, 2018) and in astroglia (Bazargani & Attwell, 2016; Verkhratsky & Kettenmann, 1996), and their ligand‐independent persistent activity has long been known documented (Ango et al., 2001). The inhibiting action of the group 1 mGluR blockade on basal Ca^2+^ indicates that, by acting either directly or indirectly on the axons under study, these receptors maintain an additional constant source of internal presynaptic Ca^2+^, be it a Ca^2+^ channel or internal Ca^2+^ store leaking Ca^2+^, a reduced capacity or affinity of the Ca^2+^ pump, or else.

Interestingly, group 1/2 mGluR blockade also reduced the spike‐evoked Ca^2+^ entry. Because the contributing role of presynaptic Ca^2+^ stores to presynaptic Ca^2+^ entry has long been demonstrated (Emptage, Reid, & Fine, 2001; Galante & Marty, 2003; Shimizu et al., 2008; Sylantyev, Jensen, Ross, & Rusakov, 2013), the possible mechanism of receptor action could be related to their well‐documented Ca^2+^ store control. At the same time, blocking glutamate uptake, which dramatically increases extrasynaptic actions of glutamate (Asztely, Erdemli, & Kullmann, 1997; Shih et al., 2013; Zheng, Scimemi, & Rusakov, 2008) boosting its average extracellular level, also decreased evoked Ca^2+^ entry, with or without group 1/2 mGluR blocked. One plausible mGluR‐independent mechanism explaining the TBOA‐dependent decrease in presynaptic Ca^2+^ is an increase in extracellular K^+^ under prolonged TBOA application (Larsen, Holm, Vilsen, & MacAulay, 2016; Lebedeva, Plata, Nosova, Tyurikova, & Semyanov, 2018; Shih et al., 2013), which would depolarize axonal terminals thus altering the contribution of axonal Na^+^ and K^+^ channels to Ca^2+^ entry (Scott et al., 2014). Intriguingly, unlike mGluR blockade, TBOA application had no effect on the basal presynaptic Ca^2+^ level. This observation lends support to the hypothesis that the mGluR‐dependent sustained source of presynaptic Ca^2+^ is not sensitive to glutamate‐receptor binding. The underpinning molecular mechanism of this functional dichotomy remains an open question.

Finally, we have found that transient (500 ms long) somatic depolarization of cortical pyramidal cells, which should mimic subthreshold excitation of the cell, does not consistently affect [Ca^2+^]_0_ or Δ[Ca^2+^] in their axons (Figure 4). Previous studies in cortical pyramidal cells and hippocampal granule cells have shown that somatic depolarization enhances release probability in their axons (Alle & Geiger, 2006; Christie et al., 2011; Scott et al., 2008; Shu et al., 2006). However, in the hippocampus, subthreshold somatic excitation had no effect on Δ[Ca^2+^] in remote (giant) boutons (Scott et al., 2008) although it did inhibit Δ[Ca^2+^] in proximal axonal segments (Ruiz et al., 2003; Scott et al., 2014). In contrast, in cortical pyramidal cells, the fluorescent intensity readout of intracellular Fluo‐5F (*K*_d_ ~ 2.3 µM) (Christie et al., 2011), or indirect tests with Ca^2+^ chelators in the presynaptic cells (Shu et al., 2006), led to a conclusion that somatic depolarization should boost Δ[Ca^2+^]. It might be important to establish reasons for the disparity between the present data and the previous observations.

## CONFLICT OF INTEREST

The authors declare no known conflict of interest.
